# Availability of ENT Surgical Procedures and Medication in Low-Income Nation Hospitals: Cause for Concern in Zambia

**DOI:** 10.1155/2020/1980123

**Published:** 2020-03-20

**Authors:** Lufunda Lukama, Chester Kalinda, Warren Kuhn, Colleen Aldous

**Affiliations:** ^1^University of KwaZulu-Natal, College of Health Sciences, Nelson R. Mandela School of Clinical Medicine, Discipline of Otorhinolaryngology, Durban 4001, South Africa; ^2^University Teaching Hospitals, Private Bag RW1X, Ridgeway, Lusaka, Zambia; ^3^University of Namibia, Katima Mulilo Campus, Private Bag 1096, Katima Mulilo, Namibia; ^4^University of KwaZulu-Natal, College of Health Sciences, Howard College Campus, School of Nursing and Public Health, Durban 4001, South Africa; ^5^University of KwaZulu-Natal, College of Health Sciences, Nelson R. Mandela School of Clinical Medicine, Durban 4001, South Africa

## Abstract

**Background:**

Ear, nose, and throat (ENT) diseases are an oft overlooked global health concern. Despite their high prevalence and associated morbidity and mortality, ENT diseases have remained neglected in health care delivery. In Zambia and many other low-income countries, ENT services are characterized by poor funding, unavailable surgical procedures, and erratic supply of essential drugs.

**Objective:**

To investigate ENT service provision in Zambia with regard to availability of surgical procedures and supply of essential drugs.

**Methods:**

A descriptive cross-sectional survey was conducted using a piloted structured questionnaire between 17 January 2017 and 2 January 2018. Included in the study were the 109 hospitals registered with the Ministry of Health (MoH) across the 10 provinces of Zambia.

**Results:**

Of the participating hospitals, only 5.9% (*n* = 1) and 40% (*n* = 1) and 40% (*n* = 1) and 40% (*n* = 1) and 40% (*n* = 1) and 40% (*n* = 1) and 40% (*n* = 1) and 40% (*n* = 1) and 40% (*n* = 1) and 40% (*n* = 1) and 40% (*n* = 1) and 40% (*n* = 1) and 40% (

**Conclusion:**

ENT service delivery in Zambia is limited with regard to performed surgical procedures and availability of essential drugs, necessitating urgent intervention. The findings from this study may be used to direct national policy on the improvement of provision of ENT services in Zambia.

## 1. Introduction

Ear, nose, and throat (ENT) diseases are common, constituting up to 50% of paediatric general practitioner (GP) consultations [1, 2]. They are a major cause of morbidity and mortality owing to the complexity and importance of the head and neck region. Hearing loss, the most common sensory deficit, disables about 5% of the world's population [3, 4]. With 400,000 to 600,000 new cases and causing 223,000 to 300,000 global deaths annually, head and neck cancer is among the world's leading malignancies [5]. Sound management of ENT diseases requires adequate resources and equipment to reduce their associated mortality and morbidity.

In many low-income countries, the burden of ENT diseases has not been matched with the resources required to effectively manage it [6–8]. ENT services are challenged by relatively small health budgets, inadequate infrastructure and equipment, shortage of medical personnel [6, 9–12], and a lack of political will [13]. As a result, fewer low-income country hospitals can manage ENT conditions than those in high-income countries [14].

Zambia, a sub-Saharan African country with a population of 16,887,720 [15], shares many ENT service provision challenges with low-income countries [6, 7, 9, 16]. Although earlier studies had drawn attention to some of the deficits in the Zambian ENT services [6, 10, 12, 17], they did not adequately explore the services with regard to performed ENT surgical procedures and drug supply to Zambian hospitals at all levels of health care, including primary, secondary, and tertiary hospitals. The country's ENT service remains among the least resourced surgical disciplines for which the government has prioritised to address in its latest National Health Strategic Plan (NHSP) [12, 18]. This study, therefore, documented the ENT surgical procedures done and medication stocked in hospitals in Zambia to highlight the deficits in the ENT service. The findings of the study may be essential in influencing the national policy on the improvement of ENT service provision in Zambia.

## 2. Methods

In this study, we followed the methods of Lukama et al. [12]. A descriptive cross-sectional survey was carried out on 109 Ministry of Health (MoH) registered hospitals across all the 10 provinces of Zambia using a structured questionnaire (Supplementary [Supplementary-material supplementary-material-1]). The survey was conducted from 17 January 2017 to 2 January 2018. The questionnaire was designed using formats adopted from previous studies [7, 19, 20] and modified to be applicable to the Zambian health system. It was trialled and validated by 5 ENT consultants within the Durban metropolitan area in the province of KwaZulu-Natal in South Africa. The first part of the questionnaire included demographic characteristics of the hospital and the level of health care of the facility. The second part focused on various ENT services with regard to the surgical procedures performed and supply of essential drugs to the hospitals. Distribution of the questionnaire to hospitals was done by post, email, or hand delivery [12].

Respondents, who were the study participants, were Medical Officers-in-Charge (MOIC) or the most appropriate health workers with knowledge of the ENT service of the individual hospitals, whose contact details were retrieved from the Zambia MoH headquarters in Lusaka. Information regarding performed surgeries and available medication at the hospitals was supplied by the relevant clinicians, pharmacists, and pharmacy technicians of the hospitals where appropriate. Where necessary, medication and nature of surgeries performed were confirmed with the authors by email and video conference calls. The participants were telephonically and by email reminded a minimum of three times to return the completed questionnaires.

Participation in the study was voluntary. All participants gave written consent. Ethical clearance was obtained from the University of KwaZulu-Natal Research Ethics Committee (Ref: BREC 463/16) and the Zambia National Health Research Authority (Ref: MH/101/23/10/1).

The collected data was entered into an electronic spreadsheet and checked for accuracy and consistency prior to analysis. The data was descriptively analysed using SPSS version 25 (IBM Corp. Released 2016. IBM SPSS Statistics for Macintosh, version 25.0, Armonk, NY: IBM Corp.). In addition to textual descriptions, results were summarized in the form of tables.

The manuscript was written using the STROBE checklist for cross-sectional studies [21] (Supplementary [Supplementary-material supplementary-material-1]).

## 3. Results

Of the 109 hospitals included in the study, 56.0% (*n* = 61) participated, comprising 83.3% (*n* = 5) of the 6 Third-Level Hospitals (TLH), 89.5% (*n* = 17) of the 19 Second-Level Hospitals (SLH), and 41.7% (*n* = 35) of the 84 First-Level Hospitals (FLH). However, 3.7% (*n* = 4) of hospitals that participated were not classified as either FLH, SLH, or TLH by the respondents. Of the participating hospitals, 62.3% (*n* = 38) were public; 18% (*n* = 11), private; and 19.7% (*n* = 12) faith-based organisation (FBO) owned. The majority of the participating hospitals (26.2%, *n* = 16) were from Lusaka Province while the least (3.3%, *n* = 2) were from Muchinga and Luapula Provinces.

### 3.1. ENT Surgical Procedures

Extraction of foreign bodies from the ears was the most widely performed ear procedure (98.4%, *n* = 60); tympanoplasty (8.1%, *n* = 5) and fitting of hearing aids (6.6%, *n* = 4) were the least performed. None of private hospitals performed tympanoplasty, mastoidectomy, or fitting of hearing aids (Tables 1 and 2).

Tables 3 and 4 detail the performance of the different nose and sinus procedures in the surveyed hospitals. Extraction of foreign bodies from the nose was the most widely performed ear procedure across all levels of hospitals.

Although all the surveyed THL carried out tracheostomy (100%, *n* = 5), only 20% (*n* = 1) and 40% (*n* = 2) performed laryngectomy and neck dissection, respectively (Table 5). None of the private hospitals carried out laryngectomy or neck dissection. However, 45.5% (*n* = 5) of private hospitals offered tracheostomy (Table 6).

### 3.2. Reasons for Nonperformance of Procedures and Surgeries by Hospitals

Inadequate equipment and absence of competent staff were the main reasons given for failure to carry out ENT surgical procedures. We observed that 75% (*n* = 12) of the SLH and 100% (*n* = 3) of the TLH suggested both lack of competent personnel and inadequate equipment as the main reasons for the failure to perform M+G. For the same reasons, 76.5% (*n* = 13) of the SLH and 75% (*n* = 3) of the TLH did not perform endoscopic sinus surgery (ESS) and 87.5% (*n* = 14) of the SLH and 66.7% (*n* = 2) of the TLH were unable to carry out laryngectomy.

### 3.3. Medication for Ear Conditions

All public, private, and FBO-owned hospitals stocked erythromycin, ciprofloxacin, amoxicillin, and ceftriaxone. In addition, all private hospitals stocked quinolone/other antibiotic ear drops. Overall, 14.8% (*n* = 9) of the surveyed hospitals stocked no antibiotic ear drops of any kind, 26.2% (*n* = 16) lacked quinolone ear drops, and 36.1% (*n* = 22) had no acetic acid (or other antiseptic ear drops). Results also showed that 68.2% (*n* = 15) of the hospitals not stocking acetic acid ear drops were public, 18.2% (*n* = 4) were private, and 13.6% (*n* = 3) were FBO owned. Of those hospitals lacking quinolone ear drops, 68.8% (*n* = 11) were public while 31.3% (*n* = 5) were FBO owned. Further, six of the nine (66.7%) hospitals not stocking non-quinolone antibiotic ear drops were public and three (33.3%) were FBO owned.

Most of the hospitals that did not stock ear drops considered the medication either unnecessary or unaffordable. Of the hospitals not stocking acetic acid ear drops, 40% (*n* = 6) public, 50% (*n* = 2) private, and 33% (*n* = 1) FBO-owned hospitals considered the medication unnecessary while 33.3% (*n* = 5) public and 33.3% (*n* = 1) FBO-owned hospitals indicated that it was unaffordable. The unavailability of quinolone ear drops was blamed on lack of necessity by 9.1% (*n* = 1) of public and 40% (*n* = 2) of FBO-owned hospitals. On the other hand, 72.7% (*n* = 8) of public and 40% of FBO hospitals considered quinolone ear drops unaffordable.

### 3.4. Medication for Sinonasal Diseases

With regard to medication for sinonasal diseases, 13.1% (*n* = 8) of the surveyed hospitals stocked leukotriene receptor antagonists, 16.4% (*n* = 10) stocked gauze impregnated with bismuth-iodoform-paraffin-paste (BIPP), and 18.0% (*n* = 11) had mast cell stabilizer nasal sprays. All private hospitals stocked topical nasal decongestants, nasal steroid sprays, chlorpheniramine, and prednisolone. In contrast, of the public hospitals, only 34.2% (*n* = 13) stocked topical nasal decongestants, 13.2% (*n* = 5) stocked nasal steroid sprays, 84.2% (*n* = 32) stocked chlorpheniramine, and 97.2% (*n* = 37) stocked prednisolone. The least stocked drugs for sinonasal diseases were leukotriene receptor antagonists (13.1%) and gauze impregnated with BIPP (16.4%).

### 3.5. Medication for Throat Diseases

All hospitals stocked benzylpenicillin, metronidazole, and injectable corticosteroids. Furthermore, 98.2% (*n* = 60) of hospitals stocked penicillin V and chloramphenicol. Only 44.7% (*n* = 17) of public, 27.3% (*n* = 8) of private, and 33.3% (*n* = 4) of FBO-owned hospitals stocked clindamycin. Of the hospitals that did not stock clindamycin, 19.4% (*n* = 6) did not consider the drug necessary and 61.3% (*n* = 19) could not afford it while 16.1% (*n* = 5) reported that the drug was both unnecessary and unaffordable. In addition, 6.5% (*n* = 2) had no knowledge about the drug.

### 3.6. Hospital Budget for ENT

None of the hospitals in Lusaka, Eastern, North-Western, and Central Provinces had a budget for ENT. Countrywide, only four hospitals (all public) had a specific budget for ENT. Of these, two were TLH (one on the Copperbelt and one in Southern Province), one was a SLH, and the other was a FLH (both in Western Province).

### 3.7. Importance of ENT to Clinical Practice

Of the study respondents, 96.7% (*n* = 59) acknowledged that ENT was an important branch of clinical practice. However, only 6.6% (*n* = 4) thought ENT had received enough consideration. In addition, 95.1% (*n* = 58) of the respondents thought ENT service delivery needed to be improved. Of these respondents, 63.8% (37/58) were from public hospitals, 19.0% (*n* = 11/58) from private hospitals, and 17.2% (*n* = 10/58) from FBO hospitals. Of the respondents who thought that ENT had not received attention, 62.5% (35/56) were from public hospitals, 17.9% (*n* = 10/56) from private hospitals, and 19.6% (*n* = 11/56) from FBO-owned hospitals.

## 4. Discussion

The status of ENT services in Zambia has not been thoroughly investigated. To the best of our knowledge, this is the first study that has comprehensively explored the availability of various ENT surgical procedures and supply of essential drugs in hospitals in Zambia at primary, secondary, and tertiary levels of health care. This study provides useful insight into the state of hospitals at all levels of care that can be used to influence policy. The results from this study indicate a lack of specialized ENT surgical services in most hospitals in Zambia leading to potential suboptimal management of ENT diseases.

A recent investigation into ENT service provision in hospitals in Zambia by Lukama et al. documented absolute deficiencies in infrastructure, human resource, and equipment in hospitals at all levels of health care [12]. The observed lack of basic ENT surgical equipment reported in this study partly accounts for the inability of most of the hospitals to offer many of the ENT surgical procedures in the country. This may lead to increased preventable morbidity and mortality from ENT diseases [18, 22, 23]. However, Lukama et al. did not investigate ENT service provision in Zambia with regard to availability of surgical procedures and supply of essential drugs, which are crucial in defining good service provision to communities.

The referral pathway for the health care service in Zambia ascends from health centres, clinics, FLH, SLH, and TLH. In general, patients are filtered through this pathway to access appropriate ENT, audiology, and speech therapy services. Accordingly, FLH are meant to treat the most basic, TLH are meant to treat the most advanced, and SLH are meant for intermediate services [13, 22]. In this study, 83.3% of TLH and 89.5% of SLH participated, representing most of the hospitals designed to offer significant ENT services. Effectively, the 41.7% participation of FLH is likely to have little impact in the results of the study.

M+G, tympanoplasty, and mastoidectomy are important surgeries in the provision of hearing health and addressing preventable hearing loss from otitis media (OM). Results from this study indicate that few hospitals carry out these surgeries, suggesting potential risk of increased incidence of OM-related hearing impairment. Emerging evidence suggests a global prevalence of OM of 30.82 per 10,000 population and an annual mortality of 21,000 [2, 22]. According to Hapunda-Chibanga [24], lack of access to these ear surgeries may have contributed to increased hearing loss among school-going children in some parts of Lusaka. We have shown that despite the high prevalence of hearing loss, access to hearing aids in low-income countries like Zambia is limited [6, 17, 25]. As a result, many people suffering from hearing loss are at risk of hearing impairment-associated morbidity such as impaired speech and language development, social isolation, and loss of potential income [4, 26].

Frontal trephination, external ethmoidectomy, and endoscopic sinus surgery (ESS) are lifesaving surgeries done to treat complicated and chronic nose and sinus disease. With 70% (*n* = 7) of provinces having no hospital to carry out these procedures, complications (orbital sepsis, brain abscesses, blindness, and death) from sinus infections and tumors are likely to be high among the Zambian population.

We have also shown that surgery to excise ENT-related tumors is inadequate in Zambia. Thus, patients who have excisable cancers may be sent for chemotherapy and radiation even when surgery offers the best chance of cure, violating sound oncological principles.

Lack of availability of ENT surgeries is a global low-income nation problem. A recent survey of 22 African countries, including Zambia, found that 66% of them had either poor or no sinus and rhinologic surgery, 87% had poor or no otologic surgery, and 75% had no or poor head and neck oncologic surgery [6]. Only a few centres, located mainly in the urban settings, offered ENT surgeries. Many factors have contributed to this problem, including shortage of ENT health personnel, lack of equipment, and political will [6, 9, 13].

This study has found that most hospitals lack essential medication for ENT conditions as listed by the World Health Organization [27, 28]. The shortage of antibiotic/antiseptic ear drops may worsen the preexisting high incidence of complications of ear infections, especially in patients attending public hospitals. In addition to the potentially devastating morbidity (hearing loss, brain abscess, and hydrocephalus) of complicated ear infections on patients, the cost of managing these complications is high. Thus, resources are diverted to managing complications instead of being fed into their preventive strategies like stock of basic medication and instruments for ear procedures. The lack of medication in hospitals may encourage potentially harmful self-medication among patients [29, 30]. In addition, the study observed that the lack of BIPP or other antiseptic nose packing material in hospitals may lead to the use of petroleum jelly-impregnated gauze to pack nose bleeds in patients. This practice may increase the patient's chances of developing sinusitis from nasal packing.

The absence of an ENT-specific hospital budget in most of the hospitals in Zambia confirms the acknowledged lack of focus given to this discipline of medical practice. Like many other low-income countries, Zambia has previously directed its national health resources towards infectious and noncommunicable resources. Recently, however, the government launched its 5-year strategic plan aimed at increasing the country's ENT infrastructure and health personnel and enhancing ENT disease public awareness [18].

To increase the availability of ENT surgeries, there needs to be a vibrant deliberate policy to train more ENT health personnel and stock necessary ENT equipment and medication. To achieve this, the country should engage high-income countries and establish collaborative programmes, a strategy which many African countries have successfully adopted [6]. Furthermore, ENT services should be decentralized to reach more people in the rural settings to effectively reduce the ENT disease-related morbidity and mortality. Leaving ENT surgeries centralized to a few urban specialist units, as is currently the case, increases waiting and patient travelling times and blocks theatre time with short-day case operations preventing complex surgeries [31]. Salisu and Jibril [32] have recently shown that providing endoscopic ear surgery in the rural settings leads to effective performance of the bulk of ear surgeries needing magnification in these places, eliminating the costs associated with patient transportation to tertiary centres and long waiting queues.

The limitation to this study was in getting feedback from nonpublic and nonurban hospitals. Like many Third World nations, Zambia's communication network is poor. Many rural places did not have a reliable Internet, and postal services were often unreliable. Some private and FBO-owned hospitals declined to participate citing legal implications. At the time of the survey, the 2012 List of Health Facilities in Zambia, released by MoH in 2013, was the latest formal publication of the list of health facilities. Therefore, tracing newly established health facilities was difficult.

Due to the shortage of ENT health personnel, some respondents to the questionnaire may not have been accurate in their reporting of procedures as they may not have been conversant with them. In addressing this, medication and nature of surgeries performed were confirmed with the authors by email and video conference calls whenever necessary.

## 5. Conclusions

ENT service delivery in Zambia remains poor with regard to performed surgical procedures and availability of essential drugs. Effective collaboration between the Zambian government and high-income countries, decentralization of health services, and government-led deliberate policy to increase ENT funding are urgently required to improve Zambia's ENT service delivery. Findings from this study are useful in directing national policy on the improvement of provision of ENT services in Zambia.

## Figures and Tables

**Table 1:** Absolute counts and percentages of the surveyed hospitals offering the different ear procedures according to levels of hospital care (first, second, and third levels).

Procedure	First-Level Hospital (FLH) (*n* = 35)	Second-Level Hospital (SLH) (*n* = 17)	Third-Level Hospital (TLH) (*n* = 5)	Unclassified (*n* = 4)	Total (*n* = 61)
Foreign body extraction	34 (97.1%)	17 (100%)	5 (100%)	4 (100%)	60 (98.4%)
Myringotomy and grommet insertion	2 (5.7%)	1 (5.9%)	2 (40%)	1 (25%)	6 (9.8%)
Tympanoplasty	2 (5.7%)	1 (5.9%)	2 (40%)	0	5 (8.2%)
Mastoidectomy	1 (2.9%)	3 (17.6%)	2 (40%)	0	6 (9.8%)
Fitting of hearing aids	1 (2.9%)	1 (95.9%)	2 (40%)	0	4 (6.6%)

**Table 2:** Absolute counts and percentages of the surveyed hospitals offering the different ear procedures according to hospital status (public, private, and faith-based organisation owned).

Procedure	Public hospital (*n* = 38)	Private hospital (*n* = 11)	Faith-based organisation-owned hospital (*n* = 12)	Total (*n* = 61)
Foreign body extraction	37 (97.4%)	11 (100%)	12 (100%)	60 (98.4%)
Myringotomy and grommet insertion	3 (7.9%)	1 (9.1%)	2 (16.7%)	6 (9.8%)
Tympanoplasty	3 (7.9%)	0	2 (16.7%)	5 (8.2%)
Mastoidectomy	5 (13.2%)	0	1 (8.3%)	6 (9.8%)
Fitting of hearing aids	3 (7.9%)	0	1 (8.3%)	4 (6.6%)

**Table 3:** Absolute counts and percentages of the surveyed hospitals offering the different nose and sinus procedures according to levels of hospital care (first, second, and third levels).

Procedure	First-Level Hospital (FLH) (*n* = 35)	Second-Level Hospital (SLH) (*n* = 17)	Third-Level Hospital (TLH) (*n* = 5)	Unclassified (*n* = 4)	Total (*n* = 61)
Extraction of foreign bodies	33 (94.3%)	17 (100%)	5 (100%)	4 (100%)	59 (96.7%)
Septoplasty	1 (2.9%)	1 (5.9%)	2 (40%)	0	4 (6.6%)
Rhinoplasty	0	1 (5.9%)	1 (20%)	0	2 (3.3%)
Frontal trephination	1 (2.9%)	2 (11.7%)	2 (40%)	1 (25%)	5 (8.2%)
External ethmoidectomy	1 (2.9%)	2 (11.7%)	2 (40%)	1 (25%)	6 (9.8%)
Endoscopic sinus surgery	1 (2.9%)	0	1 (20%)	1 (25%)	3 (4.9%)

**Table 4:** Absolute counts and percentages of the surveyed hospitals offering the different nose and sinus procedures according to hospital status (public, private, and faith-based organisation owned).

Procedure	Public hospital (*n* = 38)	Private hospital (*n* = 11)	Faith-based organisation-owned hospital (*n* = 12)	Total (*n* = 61)
Extraction of foreign bodies	36 (94.75)	11 (100%)	12 (100%)	59 (96.7%)
Septoplasty	3 (7.9%)	0	1 (8.3%)	4 (6.6%)
Rhinoplasty	2 (5.3%)	0	0	2 (3.3%)
Frontal trephination	3 (7.9%)	1 (9.1%)	1 (8.3%)	5 (8.2%)
External ethmoidectomy	3 (7.9%)	2 (18.2%)	1 (8.3%)	6 (9.8%)
Endoscopic sinus surgery	1 (2.6%)	1 (9.1%)	1 (8.3%)	3 (4.9%)

**Table 5:** Absolute counts and percentages of the surveyed hospitals offering the different head and neck procedures according to levels of hospital care (First-, Second-, and Third-Level Hospitals).

Procedure	First-Level Hospital (FLH) (*n* = 35)	Second-Level Hospital (SLH) (*n* = 17)	Third-Level Hospital (TLH) (*n* = 5)	Unclassified (*n* = 4)	Total (*n* = 61)
Fine needle aspiration cytology	2 (5.7%)	5 (29.4%)	3 (60%)	0	10 (16.4%)
Tonsillectomy	6 (17.1%)	4 (23.5%)	4 (80%)	3 (75%)	17 (27.9%)
Adenoidectomy	6 (17.1%)	4 (23.5%)	4 (80%)	3 (75%)	17 (27.9%)
Tracheostomy	8 (22.9%)	10	5 (100%)	1 (25%)	24 (39.3%)
Maxillectomy	0	0	2 (40%)	0	2 (3.3%)
Laryngectomy	0	0	1 (20%)	0	1 (1.6%)
Parotidectomy	1 (2.9%)	8 (47.1%)	4 (80%)	0	13 (21.3%)
Head and neck cancer excision	1 (2.9%)	6 (35.3%)	4 (80%)	0	11 (18.0%)
Neck dissection	0	5 (29.4%)	2 (40%)	0	7 (11.4%)
Reconstruction of defects	0	0	1 (20%)	0	1 (1.6%)

**Table 6:** Absolute counts and percentages of the surveyed hospitals offering the different head and neck procedures according to hospital status (public, private, and faith-based organisation owned).

Procedure	Public hospital (*n* = 38)	Private hospital (*n* = 11)	Faith-based organisation-owned hospital (*n* = 12)	Total (*n* = 61)
Fine needle aspiration cytology	6 (15.8%)	1 (9.1%)	3 (25%)	10 (16.4%)
Tonsillectomy	7 (18.4%)	8 (72.7%)	2 (16.7%)	17 (27.9%)
Adenoidectomy	7 (18.4%)	8 (72.7%)	2 (16.7%)	17 (27.9%)
Tracheostomy	16 (42.1%)	5 (45.5%)	3 (25%)	24 (39.3%)
Maxillectomy	2 (5.3%)	0	0	2 (3.3%)
Laryngectomy	1 (2.6%)	0	0	1 (1.6%)
Parotidectomy	10 (26.3%)	2 (18.2%)	1 (8.3%)	13 (21.3%)
Head and neck cancer excision	8 (21.1%)	61 (9.1%)	2 (16.7%)	11 (18.0%)
Neck dissection	6 (15.8%)	0	1 (8.3%)	7 (11.4%)
Reconstruction of defects	1 (2.6%)	0	0	1 (1.6%)

## Data Availability

The presented data has been archived and stored at University of KwaZulu-Natal and can be requested by following the guidelines laid in the Data Access Policy of the University of KwaZulu-Natal.

## Abbreviations

BIPP: Bismuth-iodoform-paraffin-paste
ENT: Ear, nose, and throat
ESS: Endoscopic sinus surgery
FBO: Faith-based organisation
FLH: First-Level Hospital
GP: General practitioner
M+G: Myringotomy and grommet
MoH: Ministry of Health
MOIC: Medical Officer-in-Charge
NHSP: National Health Strategic Plan
OAE: Otoacoustic emission
OM: Otitis media
SLH: Second-Level Hospital
TLH: Third-Level Hospital.

## Supplementary Materials

Supplementary Materials Supplementary file 1: the data collection tool, Hospital Survey Questionnaire, includes respondents' information, consent form, questionnaire, and description of terms. Supplementary file 2: STROBE checklist, the checklist used to write this manuscript.
