# Corrigendum to “Availability of ENT Surgical Procedures and Medication in Low-Income Nation Hospitals: Cause for Concern in Zambia”

**DOI:** 10.1155/2022/9858024

**Published:** 2022-10-14

**Authors:** Lufunda Lukama, Chester Kalinda, Warren Kuhn, Colleen Aldous

**Affiliations:** ^1^University of KwaZulu-Natal, College of Health Sciences, Nelson R. Mandela School of Clinical Medicine, Discipline of Otorhinolaryngology, Durban 4001, South Africa; ^2^University Teaching Hospitals, Private Bag RW1X, Ridgeway, Lusaka, Zambia; ^3^University of Namibia, Katima Mulilo Campus, Private Bag, 1096 Katima Mulilo, Namibia; ^4^University of KwaZulu-Natal, College of Health Sciences, Howard College Campus, School of Nursing and Public Health, Durban 4001, South Africa; ^5^University of KwaZulu-Natal, College of Health Sciences, Nelson R. Mandela School of Clinical Medicine, Durban 4001, South Africa

In the article titled “Availability of ENT Surgical Procedures and Medication in Low-Income Nation Hospitals: Cause for Concern in Zambia” [1], information was omitted in Acknowledgments.

The corrected section appears below:
