# Supplementary material for: Availability of ENT Surgical Procedures and Medication in Low-Income Nation Hospitals: Cause for Concern in Zambia
Source: Biomed Res Int. 2020 Mar 20;2020:1980123. doi: 10.1155/2020/1980123 (PMC7128045; doi:10.1155/2020/1980123)
Supplement: Supplementary Materials — Supplementary file 1: the data collection tool, Hospital Survey Questionnaire, includes respondents' information, consent form, questionnaire, and description of terms. Supplementary file 2: STROBE checklist, the checklist used to write this manuscript. [file 1980123.f1.pdf]

Supplementary file 1: Data collection tool

Hospital Questionnaire Respondents Information

Dear Health Worker,

My name is Dr Lufunda Lukama, a Medical Doctor at the University Teaching Hospital in Lusaka and Zambian Government sponsored postgraduate student at the University of KwaZulu-Natal (UKZN) in Durban.

I am conducting a survey of the ENT (Ear, Nose and Throat) Services in Zambia, a study which involves all the hospitals. The result of this survey will be used to establish the magnitude of the difficulties the ENT service delivery faces in our country and help guide future planning on how to improve the ENT health care to the Zambian people. The survey will also contribute to my Master of Medicine in ENT and Head and Neck Surgery qualification.

Permission to conduct the Survey has been granted by the Ministry of Health, Zambia and ethically reviewed and approved by UKZN Biomedical Research Ethics Committee (approval number: BE 463/16) and The University of Zambia Research Ethics Committee (approval number REF.NO.010-12-16).

I would like to kindly request you to complete the attached questionnaire and return it to the address used to deliver it to you at your earliest convenience. It takes approximately 20 to 30 minutes to complete. Your name and position at the hospital, as well as the name of the hospital will remain anonymous in the publication, which makes the risk of breach of your confidentiality negligible.

Your participation in this study is voluntary and does not come with financial or material gain. However, it would be vital in progressing our country's ENT service and ultimately improving the general health of Zambians.

For any questions and clarifications, please contact me on the address indicated below.

Regards,

Dr Lufunda Lukama, MB ChB

ENT Registrar

University Teaching Hospital, Lusaka, Zambia

University of KwaZulu-Natal, Durban, South Africa

Mobile Phones: +260 978 215191 (Zambia)

+27 72 305 0675 (South Africa)

Email: lufundal@yahoo.com

Work Address:

University of KwaZulu-Natal

College of Health Sciences

Nelson R Mandela School of Medicine

Department of Otorhinolaryngology, Head and Neck Surgery

5th Floor, Room 546, 719 Umbilo Road

Durban 4001

South Africa

Tel: +27 (0) 31 260 4292

Consent

I, ……………………………… (Name of Participant) have been informed about the study 'Survey of Ear, Nose and Throat Services in Zambia' by Dr Lufunda Lukama.

I understand the purpose, importance and cautions of the study and my questions and concerns have been satisfactorily addressed. I am aware that my participation in this study is voluntary and that my identification will remain confidential.

I understand that I may contact the researcher at the address below if I have any concerns/questions/queries related to the study.

University of KwaZulu-Natal
College of Health Sciences
Nelson R Mandela School of Medicine
Department of Otorhinolaryngology, Head and Neck Surgery
5 th Floor, Room 546
Durban 4001, South Africa
Mobile Phones:
+260 978 215191 (Zambia)
+27 72 305 0675 (South Africa)
Email: lufundal@yahoo.com

If I have any questions or concerns about my rights as a study participant, of if I am concerned about an aspect of the study or the researchers then I may contact:

UNIVERSITY OF ZAMBIA BIOMEDICAL RESEARCH ETHICS COMMITTEE
Ridgeway Campus
P.O.Bax 50110, Lusaka, ZAMBIA
Tel: +260-1-256067
Telex: UNZALU ZA 44370
Fax: +260-1-250753
Email: unzarec@unza.zm

OR

BIOMEDICAL RESEARCH ETHICS ADMINISTRATION

Research Office,Westville Campus

Govan Mbeki Building

Private Bag X 54001

Durban, 4000

KwaZulu-Natal, SOUTH FRICA

Tel: +27 31 260 4769

Fax: +27 31 260 4609

Email: BREC@ukzn.ac.za

Signature of Participant ……………………………   Date …………………

Signature of Witness ……………………………..   Date …………………

Hospital Survey Questionnaire

Hospital Code: H21

Hospital Level of Care – For Public Hospitals- (1st, 2nd or 3rd Level): ….

Hospital Catchment Population: …………………

Hospital Status: [tick as appropriate]

A. Public
B. Private
C. Faith Based Organisation Owned (Mission)…

Respondent's Position at Hospital: …………………………………

---

1. **INFRASTRUCTURE**

Do you currently have a functional …

(Tick Yes or No in or against the space provided for each option)

a) ENT dedicated examination room? **Yes** ☐ **No** ☐
b) Operating theatre? **Yes** ☐ **No** ☐
c) Operating theatre time dedicated to ENT procedures? **Yes** ☐ **No** ☐
d) Audiology Booth? **Yes** ☐ **No** ☐
e) A room or building dedicated to speech and/or language therapy?
   Yes ☐ **No** ☐
f) Functional Intensive/ Critical Care Facility? **Yes** ☐ **No** ☐
g) Functional High Dependency/High Care Facility? **Yes** ☐ **No** ☐

2. HUMAN RESOURCE

A. Core ENT Workforce

1. How many of the following Health Professions Council of Zambia (HPCZ) registered professionals do you currently have at this hospital?

(Write down the number in or against the space provided for each option)

a) Specialist (Qualified) ENT Surgeons ☐
b) Registrars and stationed in ENT ☐
c) Non-Specialist and/or Non-Registrar Medical Doctors stationed in ENT ☐
d) Medical Licentiates with further training in ENT ☐
e) Clinical Officers formally trained in ENT ☐
f) Audiologists (with a degree or higher qualification in Audiology) ☐
g) Speech therapists (with a degree or higher qualification in Speech Therapy) ☐
h) Nurses dedicated to ENT Service (e.g. stationed in the ENT consulting rooms, wards, theatres, etc.) ☐

2. What is the current total number of the following HPCZ registered professionals at this hospital?

(Write down the number in or against the space provided for each option)

a) Specialist Doctors, including ENT Surgeons ☐
b) Registrars ☐
c) Non-Specialist and/or Non-Registrar Medical Doctors ☐
d) Medical Licentiates ☐
e) Clinical Officers ☐
f) Nurses ☐

B. **Supportive Professionals**

How many of the following HPCZ registered professionals do you currently have at your facility?

(Write down the number in or against the space provided for each option)

a) Plastic and Reconstructive Surgeons ☐
b) Neurosurgeons ☐
c) Ophthalmologists (Doctors specialized in Ophthalmology) ☐
d) Maxillofacial Surgeons ☐
e) Dental Surgeons ☐
f) Vascular Surgeons ☐
g) Thoracic Surgeons ☐

3. EQUIPMENT

The following equipment relates to ENT. Please indicate how many of each item are functional at your hospital.

(Write down the number in or against the space provided for each option)

A. Basic Equipment (Clinic Equipment)

I. Ear Equipment

a) Otoscope (Auriscope) ☐
b) Pneumatic bulb for otoscope ☐
c) Tuning forks
   i. 256 Hz ☐
   ii. 512 Hz ☐
   iii. 1024 Hz ☐
d) Barany Box ☐
e) Ear syringing kit (commercial or locally assembled) ☐
f) Ear hook ☐

g) Jobson horne probe ☐

II. Nose and Sinus Equipment

a) Nasal speculum ☐

b) Nasal packing forceps ☐

c) Biopsy forceps ☐

III. Head and Neck Equipment

a) Head lamp or examination light reflecting mirror ☐

b) Laryngeal mirrors ☐

c) Tongue depressor or wooden spatula ☐

d) Suction equipment ☐

e) Fine Needle Aspiration Biopsy apparatus ☐

B. <u>Specialised Equipment</u>

1. ENT Equipment

1.1. For the Ear

a) Examination (Clinic) microscope ☐

b) Operating microscope ☐

c) Myringotomy and Grommet insertion instrument set ☐

d) Mastoid drill kit ☐

e) Tympanomastoidectomy instrument set ☐

1.2. For the Nose and Paranasal Sinuses

a. Rigid rhinoscope

i. Zero degree ☐

ii. Thirty degree ☐

iii. Forty-Five degree ☐

iv. Seventy degree ☐

b. Flexible rhinolaryngoscope ☐

c. Endoscopic Sinus Surgery Instrument Set ☐

d. Frontal trephine set ☐
e. Endoscopic microdebrider ☐
f. Bipolar forceps/Ligature clips for Sphenopalatine artery cauterization/ligation ☐

1.3. For Head and Neck

a. Stroboscope ☐
b. Tonsillectomy and Adenoidectomy set ☐
c. Tracheostomy instrument set ☐
d. Speech Valve insertion kit ☐
e. Facial Nerve Monitor ☐

2. Audiological Equipment

a. Otoscope (Auriscope) ☐
b. Ear Syringing ☐
c. Audiometer
   i. Screening ☐
   ii. Diagnostic (including bone conduction transducer) ☐
d. Tympanometer ☐
e. ABR (Auditory Brainstem Response) Equipment
   i. Screening ☐
   ii. Diagnostic ☐
f. OAE (Otoacoustic Emission) Equipment
   i. Screening ☐
   ii. Diagnostic ☐
g. Hearing Aid (HA)
   i. Computer ☐
   ii. Programmer (HI PRO) or equivalent ☐
   iii. Hearing Aid Software ☐
   iv. Hearing Aid Evaluations- impressions ☐
   v. Hearing Aid Fittings ☐

vi. Aural Rehabilitation

h. Auditory Steady State Response (ASSR) Equipment
i. Acoustic Reflex Testing Equipment
j. Electronystagmography (ENG) Equipment
k. Videonystagmography (VNG) Equipment

3. Speech Therapy Equipment and Devices

a. Fibreoptic laryngoscope for Evaluation of swallow
b. Devices
    i. Voice Prosthesis
    ii. Speaking valve
    iii. Humidifiers
    iv. Electrolarynx
    v. Trache brushes
    vi. Mirrors
    vii. BIBS
    viii. Pacifiers
    ix. Specialised feeding bottles and cups
    x. Plastic Spoons for baby feeding
c. Augmentative and Alternative Communication (ACC) Devices
d. Printer and Laminating Machines

C. Supportive Specialised Equipment

a. Fluoroscopy Equipment
b. CAT (CT) Scanner
c. MRI Scanner
d. PET Scanning Equipment
e. Radiotherapy Machine

4. **SURGICAL PROCEDURES**

Kindly indicate which of the following procedures your facility performs:

A) Procedures and Operations

Which of the following surgical procedures and operations do you perform at your facility? Indicate Yes or No against each option:

1. **For the Ear**
   a) Extraction of foreign bodies from the ear ☐
   b) Myringotomy and grommet insertion ☐
   c) Tympanoplasty ☐
   d) Mastoidectomy ☐
   e) Fitting of hearing aids ☐

2. **For the Nose and Sinuses**
   a) Extraction of foreign bodies from the nose ☐
   b) Septoplasty ☐
   c) Rhinoplasty ☐
   d) Frontal Trephination ☐
   e) External Ethmoidectomy ☐
   f) Endoscopic Sinus Surgery ☐

3. **For Head and Neck**
   a) Fine Needle Aspiration Cytology ☐
   b) Tonsillectomy ☐
   c) Adenoidectomy ☐
   d) Tracheostomy ☐
   e) Maxillectomy ☐
   f) Laryngectomy ☐

g) Parotidectomy ☐
h) Excision of Head and Neck Cancers ☐
i) Neck Dissection ☐
j) Reconstruction of defects left by excision of Head and Neck Cancers ☐

B) <u>Reasons for non-performance of the procedures and operations</u>

For the procedures and operations <u>not done</u> at your hospital, indicate against each option the reason why using the number code below:

1 for non-availability of instruments and/or equipment
2 for non-availability of competent person to do it
3 for both no equipment and a competent person to do it
4 for non-availability of theatre
5 for non-availability of Critical Care Facilities i.e. ICU
6 for both no theatre and no Critical Care Facilities
7 for non-applicable

1. **For the Ear**
   a) Extraction of foreign bodies from the ear ☐
   b) Myringotomy and grommet insertion ☐
   c) Tympanoplasty ☐
   d) Mastoidectomy ☐
   e) Fitting of hearing aids ☐

2. **For the Nose and Sinuses**
   a) Extraction of foreign bodies from the nose ☐
   b) Septoplasty ☐
   c) Rhinoplasty ☐
   d) Frontal Trephination ☐
   e) External Ethmoidectomy ☐
   f) Endoscopic Sinus Surgery ☐

3. **For Head and Neck**

a) Fine Needle Aspiration Cytology ☐
b) Tonsillectomy ☐
c) Adenoidectomy ☐
d) Tracheostomy ☐
e) Maxillectomy ☐
f) Laryngectomy ☐
g) Parotidectomy ☐
h) Excision of Head and Neck Cancers ☐
i) Neck Dissection ☐
j) Reconstruction of defects left by excision of Head and Neck Cancers ☐

5. <u>ESSENTIAL DRUGS</u>

A) Do you stock the following medication?

Indicate "**Yes"** or "**No"** against each option

1. **Medication used for Ear Diseases**
   a) Amoxicillin or Amoxiclav ☐
   b) Erythromycin ☐
   c) Ciprofloxacin ☐
   d) Ceftriaxone ☐
   e) Acetic acid or other antiseptic ear drops ☐
   f) Quinolone ear drops e.g. ciprofloxacin or ofloxacin ☐
   g) Other antibiotic ear drops ☐

2. **Medication used for Nasal Diseases**
   a) Gauze coated with BIPP (Bismuth Iodoform Paraffin Paste) or other nasal packing antiseptic paste ☐
   b) Topical Nasal decongestants e.g. xylometazoline, oxymetazoline ☐
   c) Nasal steroid sprays/drops ☐
   d) Chlorpheniramine, cetirizine, loratidine or other antihistamine (oral or injectable) ☐

e) Sodium Chromoglycate or other mast cell stabilizers nosedrops ☐

f) Ipratropium Bromide or other anticholinergic intranasal spray ☐

g) Montelukast or other Leukotriene Receptor Antagonist (oral formulation) ☐

h) Prednisolone ☐

i) Cloxacillin ☐

j) Saline or bicarbonate nasal douche/wash ☐

k) Systemic Antifungal agents e.g. Amphotericin B, Voriconazole ☐

3. Medication used for Throat Diseases

a) Benzylpenicillin (X-Pen) ☐

b) Penicillin V ☐

c) Metronidazole (oral or injectable) ☐

d) Clindamycin (oral or injectable) ☐

e) Chloramphenicol (oral or injectable) ☐

f) Injectable corticosteroids e.g. hydrocortisone, dexamethasone ☐

g) Proton pump inhibitors e.g. omeprazole, lansoprazole (injectable or oral) ☐

C) For the medication you do not stock, what is the reason for not stocking it?

Please indicate against each option with the code below:

1 for not considered necessary

2 for not affordable or expensive

3 for both not necessary and not affordable

4 for no knowledge about the drug

5 for not applicable

1. Medication used for Ear Disease

a) Amoxicillin or Amoxiclav ☐

b) Erythromycin ☐

c) Ciprofloxacin ☐

d) Ceftriaxone ☐

e) Acetic acid or other antiseptic ear drops ☐

f) Quinolone ear drops e.g. ciprofloxacin or ofloxacin ☐

g) Other antibiotic ear drops ☐

2. Medication used for Nasal and Sinus Disease

a) Gauze coated with BIPP (Bismuth Iodoform Paraffin Paste) or other nasal packing antiseptic paste ☐

b) Topical Nasal decongestants e.g. xylometazoline, oxymetazoline ☐

c) Nasal steroid sprays/drops ☐

d) Chlorpheniramine, cetirizine, loratidine or other antihistamine (oral or injectable) ☐

e) Sodium Chromoglycate or other mast cell stabilizers ☐

f) Ipratropium Bromide or other anticholinergic intranasal spray ☐

g) Montelukast or other Leukotriene Receptor Antagonist ☐

h) Prednisolone ☐

i) Cloxacillin ☐

j) Saline or bicarbonate nasal douche/wash ☐

3. Medication used for Throat Disease

a) Benzylpenicillin (X-Pen) ☐

b) Penicillin V ☐

c) Metronidazole (oral or injectable) ☐

d) Clindamycin (oral or injectable) ☐

e) Chloramphenicol (oral or injectable) ☐

f) Injectable corticosteroids e.g. hydrocortisone, dexamethasone ☐

6. BUDGET AND ENT RELEVANCE PERCEPTION

Indicate "**Yes**", "**No" or "Not Sure"** against each option

a) Do you have a hospital budget specific for ENT? ☐
b) If Yes, what proportion of the whole hospital budget is the ENT budget? Indicate as a percentage. ☐
c) Is ENT an important branch of Clinical Practice? ☐
d) Do you think the ENT service has received enough attention at your hospital? ☐
e) Do you think ENT service delivery needs to be improved at your Hospital? ☐

You have come to the end of the Questionnaire. Thank you for your time.

END OF QUESTIONNAIRE

Description of Terms

The terms described below are applicable to Zambia.

1. **Third Level Hospitals (**Specialist or Tertiary Hospitals)
   The highest referral hospitals in Zambia, catering for a catchment population of approximately 800,000 and above, and have sub-specialisations in Internal Medicine, Surgery, Paediatrics, Obstetrics and Gynaecology, Intensive Care, Psychiatry, Training and Research. All complicated cases not attended to at second level hospitals are referred to third level hospitals. (The 2012 List of Health Facilities in Zambia Preliminary Report. Zambia. 2013).
   The sub-specialisations include Nephrology, Rheumatology, Cardiology, Neurology, Radiology, Dermatology, Oncology, Infectious Disease, Endocrinology, Otorhinolaryngology, Neurosurgery, Ophthalmology, Maxillofacial Surgery, Paediatric Surgery, Neonatology, Nutrition and Dietetics. They may not be found at every Third Level Hospital.

2. **Second Level Hospitals (Provincial or General Hospitals)**
   They are intended to cater for a catchment area of between 200,000 and 800,000 people, with services in Internal Medicine, General Surgery, Paediatrics, Obstetrics and Gynaecology, Dentistry, Psychiatry and Intensive Care. They also act as referrals for the first level institutions, including the provision of technical back up and training. (The 2012 List of Health Facilities in Zambia Preliminary Report. Zambia. 2013)

3. **First level hospitals (District Hospitals)**
   They are the third highest levels of care after the Second and Third Level referral hospitals, serving a population of between 80,000 and 200,000 and providing Medical, Surgical, Obstetric and Diagnostic services and all clinical support of Health Centre referrals. (The 2012 List of Health Facilities in Zambia Preliminary Report. Zambia. 2013)

4. **Registrar**

   A medical doctor training to be a specialist and has successfully completed at least one year of the Master of Medicine training programme.

5. **Medical Licentiate**

   A clinician holding an Advanced Diploma in General Medicine or Specialty of Medicine or a Bachelor of Science in Clinical Science.

6. **Clinical Officer**

   A Clinician holding a Diploma in Clinical Medical Sciences or equivalent.

7. **Private Hospital**

   Hospital not owned by the State

8. **Faith Based Organisation**

   Religious Organisation, often referred to as a Mission

Supplementary file 2: STROBE checklist

	Item No	Page Number	Recommendation
**Title and abstract**	1	2	(*a*) Indicate the study's design with a commonly used term in the title or the abstract
		2	(*b*) Provide in the abstract an informative and balanced summary of what was done and what was found
**Introduction**			
Background/rationale	2	3-4	Explain the scientific background and rationale for the investigation being reported
Objectives	3	4	State specific objectives, including any prespecified hypotheses
**Methods**			
Study design	4	4	Present key elements of study design early in the paper
Setting	5	4-5	Describe the setting, locations, and relevant dates, including periods of recruitment, exposure, follow-up, and data collection
Participants	6	4-5	(*a*) Give the eligibility criteria, and the sources and methods of selection of participants
Variables	7	4-5	Clearly define all outcomes, exposures, predictors, potential confounders, and effect modifiers. Give diagnostic criteria, if applicable
Data sources/ measurement	8*	4-5	For each variable of interest, give sources of data and details of methods of assessment (measurement). Describe comparability of assessment methods if there is more than one group
Bias	9		Describe any efforts to address potential sources of bias
Study size	10	4	Explain how the study size was arrived at
Quantitative variables	11	4-5	Explain how quantitative variables were handled in the analyses. If applicable, describe which groupings were chosen and why
Statistical methods	12	5	(*a*) Describe all statistical methods, including those used to control for confounding
		Not applicable	(*b*) Describe any methods used to examine subgroups and interactions
		Not applicable	(*c*) Explain how missing data were addressed
		5	(*d*) If applicable, describe analytical methods taking account of sampling strategy
		Not applicable	(*e*) Describe any sensitivity analyses

		**Results**	
Participants	13*	4-5	(a) Report numbers of individuals at each stage of study—eg numbers potentially eligible, examined for eligibility, confirmed eligible, included in the study, completing follow-up, and analysed
		4-5	(b) Give reasons for non-participation at each stage
		Not applicable	(c) Consider use of a flow diagram
Descriptive data	14*	5	(a) Give characteristics of study participants (eg demographic, clinical, social) and information on exposures and potential confounders
		Not applicable	(b) Indicate number of participants with missing data for each variable of interest
Outcome data	15*	5	Report numbers of outcome events or summary measures
Main results	16	Not applicable	(*a*) Give unadjusted estimates and, if applicable, confounder-adjusted estimates and their precision (eg, 95% confidence interval). Make clear which confounders were adjusted for and why they were included
		Not applicable	(*b*) Report category boundaries when continuous variables were categorized
		Not applicable	(*c*) If relevant, consider translating estimates of relative risk into absolute risk for a meaningful time period
Other analyses	17	Not applicable	Report other analyses done—eg analyses of subgroups and interactions, and sensitivity analyses
		**Discussion**	
Key results	18	16	Summarise key results with reference to study objectives
Limitations	19	19	Discuss limitations of the study, taking into account sources of potential bias or imprecision. Discuss both direction and magnitude of any potential bias
Interpretation	20	16-20	Give a cautious overall interpretation of results considering objectives, limitations, multiplicity of analyses, results from similar studies, and other relevant evidence
Generalisability	21	16	Discuss the generalisability (external validity) of the study results
		**Other information**	
Funding	22	24	Give the source of funding and the role of the funders for the present study and, if applicable, for the original study on which the present article is based
